# Effect of an Educational Intervention on Medical Student Scripting and Patient Satisfaction: A Randomized Trial

**DOI:** 10.5811/westjem.2018.1.35992

**Published:** 2018-03-08

**Authors:** Katie E. Pettit, Joseph S. Turner, Katherine A. Pollard, Bryce B. Buente, Aloysius J. Humbert, Anthony J. Perkins, Cherri D. Hobgood, Jeffrey A. Kline

**Affiliations:** * Indiana University School of Medicine, Department of Emergency Medicine, Indianapolis, Indiana; † Washington University School of Medicine, Department of Medicine, St. Louis, Missouri; ‡ Marion University College of Osteopathic Medicine, Indianapolis, Indiana; § Indiana University Center for Health Innovation and Implementation Science, Indiana Clinical and Translational Sciences Institute, Indianapolis, Indiana

## Abstract

**Introduction:**

Effective communication between clinicians and patients has been shown to improve patient outcomes, reduce malpractice liability, and is now being tied to reimbursement. Use of a communication strategy known as “scripting” has been suggested to improve patient satisfaction in multiple hospital settings, but the frequency with which medical students use this strategy and whether this affects patient perception of medical student care is unknown. Our objective was to measure the use of targeted communication skills after an educational intervention as well as to further clarify the relationship between communication element usage and patient satisfaction.

**Methods:**

Medical students were block randomized into the control or intervention group. Those in the intervention group received refresher training in scripted communication. Those in the control group received no instruction or other intervention related to communication. Use of six explicit communication behaviors were recorded by trained study observers: 1) acknowledging the patient by name, 2) introducing themselves as medical students, 3) explaining their role in the patient’s care, 4) explaining the care plan, 5) providing an estimated duration of time to be spent in the emergency department (ED), and 6) notifying the patient that another provider would also be seeing them. Patients then completed a survey regarding their satisfaction with the medical student encounter.

**Results:**

We observed 474 medical student-patient encounters in the ED (231 in the control group and 243 in the intervention group). We were unable to detect a statistically significant difference in communication element use between the intervention and control groups. One of the communication elements, explaining steps in the care plan, was positively associated with patient perception of the medical student’s overall communication skills. Otherwise, there was no statistically significant association between element use and patient satisfaction.

**Conclusion:**

We were unable to demonstrate any improvement in student use of communication elements or in patient satisfaction after refresher training in scripted communication. Furthermore, there was little variation in patient satisfaction based on the use of scripted communication elements. Effective communication with patients in the ED is complicated and requires further investigation on how to provide this skill set.

## INTRODUCTION

The medical community has embraced the importance of sound communication in the physician-patient relationship. Effective communication has been associated with improved patient outcomes^1^,^2^ and patient satisfaction.^3^ Patient satisfaction, in turn, has become an important benchmark for many hospital systems.

Communication skills are difficult to teach, implement, and evaluate. Recent advancements in undergraduate medical curricula have sought to improve communication skills.^4^–^7^ Some medical schools have recognized communication as a competency to further emphasize development of this important skill.^8^ Despite these recent advancements, there is still a need for improvement. Research suggests that medical students, likely more focused on expanding their medical knowledge, under-appreciate the importance of communication skills in the practice of medicine.^9^

Healthcare consultants have suggested scripting as one method to improve communication with patients. Scripting has previously been shown to have a positive impact on patient satisfaction^10^,^11^ and elopement rates^12^ from the emergency department (ED). We thus undertook a previous pilot study to assess the association of scripted communication elements with patient satisfaction in the ED, an environment that presents a unique set of communication challenges, especially for novice learners.^13^

In the pilot study, we chose to use a modified version of the Studer Group’s AIDET^®^ mnemonic to teach scripted communication elements to medical students rotating through the ED. The mnemonic reminds the provider of simple communication elements: acknowledging the patient by name, introducing themselves by name, providing an expected duration, and explaining the steps in the patient’s care plan.

Our pilot study found that medical students use these targeted communication elements inconsistently, but that their use was associated with improved patient satisfaction. The low rate with which medical students used basic communication skills, such as acknowledging the patient by name, confirmed the need for additional education in this area.^13^ Based on this preliminary data, we implemented an educational intervention emphasizing scripting to improve communication.

The objectives of this study were to measure the use of targeted communication skills after a refresher educational intervention as well as to further clarify their relationship with patient satisfaction. We hypothesized that students who received the refresher training would be more likely to use scripted communication and that this would be associated with higher patient satisfaction scores.

## METHODS

### Design and Setting

This was a randomized controlled trial conducted between July 2014 and April 2015 in the EDs of two urban teaching hospitals affiliated with the Indiana University School of Medicine. The Sidney and Lois Eskenazi Hospital (Hospital A) is a county hospital with approximately 100,000 patient visits annually. Indiana University Health Methodist Hospital (Hospital B) is a tertiary referral center, also with approximately 100,000 patient visits annually. The study was approved by the Indiana University Institutional Review Board.

**Population Health Research Capsule**

What do we already know about this issue?

Effective communication in the physician-patient relationship improves patient outcomes and patient satisfaction. Scripting is a suggested method to improve these skills.

What was the research question?

Does an educational intervention improve medical student use of communication skills and improve patient satisfaction?

What was the major finding of the study?

Patient satisfaction did not improve with the use of scripted communication or the educational intervention.

How does this improve population health?

Improving communication within the physician-patient relationship is a multifactorial construct and cannot rely on scripted communication elements alone.

### Participants

Fourth-year medical students were enrolled on a volunteer basis and provided written consent at the orientation to their emergency medicine (EM) clerkship, a required 4-week clinical course at Indiana University School of Medicine. There was no incentive for participation. Study information was given and consent was obtained by an EM resident who was not responsible for their grade. Students participating in the study were informed that they would be observed while on shift in the ED but were otherwise kept blind as to what was being observed.

Patients who could provide verbal consent (>18 years old or had a parent present to consent) in English or Spanish and who were evaluated by a participating medical student were given the option to participate in a patient satisfaction survey. Surveys were not administered to patients with the following conditions: incarcerated, altered mental status, psychiatric chief complaint (suicidal ideation, homicidal ideation, aggressive behavior, depression, anxiety, or psychosis), or critical illness (unstable vital signs, respiratory distress, or triaged to the high acuity area of the ED).

### Intervention and Randomization

All students at Indiana University School of Medicine participate in a brief session introducing scripted communication prior to starting their third-year clinical rotations (13–20 months prior to participation in our study). For this study, students participating in the clerkship each month were block randomized by rotation site, using a block size of six, to receive additional refresher training on scripted communication (intervention group) or no additional training (control group). The randomization schedule is shown in Table 1. The refresher training consisted of a 10-minute video presentation about scripted communication provided on the first day of the rotation. This presentation carried the logo of the respective healthcare system and was shown to the students during their clinical site orientation rather than at the course orientation to keep students blind regarding the association of the presentation with the study and the clerkship. Students randomized to the intervention were also provided a handout emphasizing the importance of scripted communication. The control group was not provided with these materials prior to their clerkship, but they were provided with this education at the conclusion of the study.

### Outcome Measures

Six communication elements were previously chosen for observation as outlined in our pilot study.^13^ The elements are shown in Table 2. They are based on AIDET^®^, a patient communication framework by The Studer Group. We assessed patient satisfaction through the same four-part survey used in that study (Appendix A). The primary outcome of interest was change in the frequency of “yes” responses to questions about likelihood to return to the ED or likelihood to refer a loved one to the ED. Secondary outcomes of interest included frequency of use of each of the six elements, improvement in the patient’s perception of the student’s overall communication skill, and improvement in score on the Communication Assessment Tool (CAT). The CAT is a previously validated instrument that assesses interpersonal and communication skills using a 15-item survey with a five-point Likert scale (1 = poor, 2 = fair, 3 = good, 4 = very good, 5 = excellent).^14^ We modified the survey by removing one question, “The doctor’s staff treated me with respect,” to keep focus on the student-patient interaction rather than the patient’s overall experience.

### Observers and Study Procedure

Four observers, all students in the pre-medical program at Indiana University-Purdue University Indianapolis, were trained by study investigators to navigate participating EDs and record elements of patient-student interactions on a data collection form. Data collection forms included whether or not the student used each of the six communication elements as well as whether the student performed 17 additional “dummy” data points, which were chosen by study investigators as actions commonly performed by students. These were added to keep the student and observers blind to what elements were of interest for the study. Refer to Appendix B for the complete data collection sheet with all “dummy” data points.

As part of their training, the four observers viewed 31 simulated video recordings of interactions between a patient and a provider and marked whether the provider used each of the six communication elements of interest as well as whether they performed each of the 17 “dummy” data points. Responses for each of the observers were compared to “criterion standard” responses from a fifth observer, the Masters of Public Health student who had performed all observations in our previous study.^13^ We calculated agreement of the observers with the criterion standard as kappa and percent agreement.

Each month, the four observers were scheduled for a variety of shifts across multiple days and times. For each shift, the observer was assigned to follow 1–3 participating medical students. Observers followed their assigned students and completed the data collection sheet for each patient encounter.

After the student-patient encounter but before discharge or admission, the observer returned to the patient’s room and verbally administered the patient satisfaction survey. At this time, the observer presented the patient with a picture of the student and stressed that the questions applied specifically to the patient’s interaction with that student and not other aspects of the patient’s care in the ED. The satisfaction survey was done without the students’ knowledge.

Following each shift, all data from the data collection forms and associated patient satisfaction surveys were stored in RedCap.^15^ REDCap (Research Electronic Data Capture) is a secure, web-based application designed to support data capture for research studies.

### Power Analysis

The length of this study was determined by the usage of communication elements in our pilot study as well as data provided by hospital administration on expected baseline patient satisfaction. We estimated from this data that the baseline rate of “yes” responses would be between 50–60% for Hospital B and 30–40% for Hospital A. We recognized this value would fluctuate month to month, but the randomized design and the fact that intervention and control subjects would be studied in back-to-back months would help control for that variance. With 20 students rotating at the study sites per month and >100,000 visits annually at each ED, preliminary power calculation estimates with α=0.05, an effect size of 10%, change in score from 45% to 55% between groups and N=750 encounters per group yielded a power of 97%.

### Data Analysis

We used chi-square test (p<0.05 significant) to test the bivariate association of communication elements with likelihood to return, likelihood to refer, and excellent overall communication skill. Two-tailed t-tests and chi-square tests were used to determine if student characteristics differed by randomization group. We used chi-square tests to determine if the dichotomous items (each of the six communication elements, referral to ED, return to ED, and excellent overall communication) differed by randomization group, while two-tailed t-tests were used to determine if the overall CAT score differed by the intervention.

Since multiple assessments were done on each student, we also performed mixed effects regressions (logistic for dichotomous outcomes and linear for continuous outcomes) to account for repeated measures across students. For these models, intervention was included as the only fixed effect, while a random effect for student was included to account for repeated measurements across students. Additionally, we ran models adjusting for student characteristics (gender, age, intended specialty, and rotation site). Results were similar; therefore, we only report those results with no adjustment. All analyses were performed using SAS v9.4.

## RESULTS

During the simulated encounters used for observer training, there was high level of agreement between the four observers for each of the six primary data points (Appendix C).

### Demographics

Eighty medical students were observed during the eight-month study period. One student declined to participate. Forty-five of the students were male. Twenty-nine planned to pursue emergency medicine (EM), and 51 planned to pursue other specialties (including anesthesiology, family medicine, general surgery, internal medicine, neurology, neurosurgery, obstetrics-gynecology, otolaryngology, orthopedic surgery, pathology, psychiatry, radiology, other surgical specialty, other non-surgical specialty, and multiple/unsure). There was no statistically significant difference between the groups in terms of the percentage of students pursuing a specialty in EM (p = 0.062). Four hundred seventy-four medical student-patient encounters were observed (231 in the control group and 243 in the intervention group). All observations that were begun were completed. Table 3 provides additional characteristics of the observed students.

### Communication Element Use

Data for the use of communication elements in the control and intervention groups is shown in the Figure. The most frequently used element in both the control and intervention groups was the student introducing himself or herself by name, which occurred during 96.1% and 97.9% of encounters in the control and intervention groups, respectively. The least frequently used element was providing the patient with an expected duration of stay, which occurred during 11.3% and 13.1% of encounters in the control and intervention groups, respectively.

### Comparative Data

Table 4 displays the association between each of the six communication elements and patient satisfaction outcomes. Explaining steps in the care plan was associated with an increased likelihood that the patient would give the student an “excellent” rating in overall communication skills. Otherwise, there was no statistically significant association between element use and patient satisfaction.

Table 5 shows the association of the outcome measures with placement in the control or intervention groups. There were no statistically significant associations between group and outcome measures. The intervention group did receive a slightly, but not statistically significant, higher frequency of “yes” responses to the questions about likelihood to return and to refer, a higher percentage of excellent ratings in overall communication skill, and a higher mean score on the CAT.

## DISCUSSION

Our previous study demonstrated that medical student use of specific communication elements was associated with increased patient satisfaction but that medical students use these elements inconsistently.^13^ Additionally, baseline medical student use of what may be considered the most basic of communication elements – such as acknowledging the patient by name – was surprisingly low (61%) in our previous study. We therefore developed and tested an educational intervention in an attempt to increase student use of these communication elements and further explore the association of these communication elements with patient satisfaction. In contrast to our previous results, the current study found no increase in patient satisfaction with our intervention and little association between use of scripting and patient satisfaction. The single significant association between the intervention group and use of the explaining role element was possibly due to chance given the number of outcomes analyzed and lost significance in the mixed-effects model.

Interestingly, baseline medical student (non-intervention) use of all communication elements in this study was much higher than in our previous study. Such a high baseline use of scripting may have contributed to the failure of the intervention to increase usage above that baseline rate. The reason for this increased utilization is unclear. To our knowledge, medical students did not receive any new formalized communication training in comparison to the previous study group, and observer training was also unchanged. It is possible that increased emphasis on communication throughout the medical school has resulted in improved modeling of good communication by faculty and teachers, or that medical student admissions processes have adapted to address communication skills among those accepted to the school. Additionally, the higher than anticipated baseline use of elements certainly affected the power of our study as we used much lower rates in our power analysis.

Our previous study found a strong association between use of several of the communication elements and increased rates of patient satisfaction as measured by our selected outcomes. The current study did not confirm this association. Only one element-outcome pair, “Explain-Overall Communication Skill” maintained statistical significance in this study. With 18 element-outcome pairs, it is possible that this single association occurred by chance. However, the fact that this “Explain-Overall Communication Skill” pair was also significant in our pilot study raises the possibility that this represents a result of the intervention rather than a chance event.

The other statistically significant associations found in the pilot study lost their significance in the current study. Two of the significant associations from the pilot study, the “Acknowledge-Refer” and “Acknowledge-Overall Communication Skill” pairs showed a small trend toward a positive association in the current study. It is possible that significance was lost due to much higher element use across the board, making it more difficult to show a difference.

In the current study, patient satisfaction scores were not significantly improved in students randomized to our intervention. This is not surprising given the failure of the intervention to significantly increase student use of most of the scripted elements that were emphasized. Our intervention was brief, and it is possible that a more robust intervention might have increased the use of scripted elements. However, it is still unknown if this would have had a positive effect on patient satisfaction. Even if there is some effect of the use of scripted communication elements on satisfaction, our current results suggest that the magnitude of this effect seems to be small.

The most likely explanation for the failure of this study to show an association between the selected scripted communication elements and patient satisfaction is that improving patient satisfaction is a multifactorial construct and the contribution of adding scripted communication elements is very small. Using scripted communication as a strategy to improve patient satisfaction is only a small piece of a much larger puzzle. Scripted communication may help providers remember a baseline level of communication, and this study does not indicate that initial training in scripted communication is not valuable. However, our study indicates refresher training in scripting itself is not enough to improve communication beyond a baseline level. The effect of refresher training and of scripted communication in general may also be influenced by experience and level of training, and it is possible that different results would be obtained with different levels of providers. Future research should focus beyond a simple communication checkbox. Perhaps there would be benefit with interventions that help providers better understand the patient’s perspective, experience, and expectation.

## LIMITATIONS

There were several limitations to this study. The study group consisted of a sample of medical students from a single medical school. While we attempted to blind the students to the nature of the study, the Hawthorne effect resulting from the knowledge that they were being observed may have contributed to increased use of all communication elements in both groups, limiting our ability to show a difference between groups. Also, while we took measures to avoid the intervention group influencing the control group (such as holding the intervention at clinical site orientation rather than the clerkship orientation), there is no guarantee that the groups did not communicate about the intervention.

Additionally, the study is limited by the lack of explicit testing of the validity of the outcome measures. The patient satisfaction survey is similar to actual surveys that are widely used in hospital systems like ours, and the CAT tool has been previously validated for other purposes. However, both tools were modified for the purposes of our study, which could threaten their validity. Finally, although we stressed to the patient that the survey pertained only to their encounter with the student, it is possible that other aspects of their visit – including interactions with other providers – influenced survey results. It is also likely that other unmeasured verbal and non-verbal aspects of communication may have influenced results. We were also not able to control medical student exposure to other forms of communication education and did not examine medical student retention of the information covered during our education intervention.

## CONCLUSION

We hypothesized that an educational intervention to increase use of scripted communication elements would result in increased patient satisfaction. Unfortunately, our intervention did not result in any increase in either use of scripting by students or patient satisfaction. Additionally, this study failed to confirm earlier findings of an association between scripted communication elements and patient satisfaction. Communicating effectively with patients is likely much more complex than using a sample of scripted communication elements, and further research on optimizing patient-provider communication is urgently needed.

## Supplementary Information







## Figures and Tables

**Figure:**
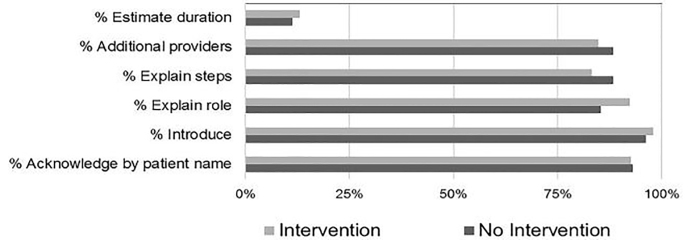
Rate of communication element use by group.

**Table 1:** Randomization by site of med students participating in research on scripted communication with patients.

	Hospital A	Hospital B
July 2014	Intervention	Intervention
August 2014	Intervention	Control
September 2014	Control	Intervention
November 2014	Control	Control
January 2015	Intervention	Control
February 2015	Control	Intervention

**Table 2:** Observed communication elements.

Did the student acknowledge the patient using the patient’s name?
Did the student introduce himself/herself by name?
Did the student explain his/her role as a medical student?
Did the student explain some of the steps (including diagnostic testing, medication administration, or observation) that would be used to address the patient’s complaint?
Did the student explain that additional providers (such as a resident or attending physician) would also be evaluating the patient?
Did the student offer an estimated duration of time that the patient would spend in the ED?†

† For estimated duration, a general statement of time (e.g.,“overnight” or “a few hours”) was considered acceptable; a specific number was not required.

**Table 3:** Characteristics of med students who participated in an eight-month study of patient satisfaction with student communication.

	Control (n=40)	Intervention (n=40)	P value
Site			1.000
% Hospital A (n)	55.0 (22)	55.0 (22)	
% Hospital B (n)	45.0 (18)	45.0 (18)	
% Male (n)	52.5 (21)	60.0 (24)	0.652
% Emergency medicine (n)	25.0 (10)	47.5 (19)	0.062
Mean age (SD)	26.6 (2.6)	26.6 (1.6)	0.628

*SD,* standard deviation.

**Table 4:** Association of element use with patient satisfaction outcomes.

	Student encounter would make patient choose ED again (%)	Student encounter would make patient refer a loved one to the ED (%)	% Rate student’s overall communication skill = 5 (Excellent)
Student did not acknowledge patient by name (n=34)	91.2	91.2	76.5
Student acknowledge patient by name (n=440)	91.5	96.1	85.9
P-value	0.320	0.194	0.193
Student did not introduce themselves by name (n=14)	100.0	100.0	85.7
Student introduced themselves by name (n=460)	94.8	95.6	85.0
P-value	0.796	0.903	0.928
Student did not describe role as a medical student (n=53)	96.2	96.2	84.9
Student described role as a medical student (n=422)	94.8	95.7	85.1
P-value	0.657	0.868	0.995
Student did not explain any steps in care plan (n=67)	95.5	95.5	73.1
Student explained some steps in care plan (n=403)	94.8	95.8	86.8
P-value	0.802	0.923	0.010
Student did not explain other providers would see patient (n=64)	95.3	95.3	82.8
Student explained other providers would see patient (n=411)	94.9	95.9	85.4
P-value	0.887	0.840	0.578
Student did not provide estimated duration (n=410)	94.6	95.4	86.1
Student provided estimated duration (n=57)	96.5	98.3	77.2
P-value	0.559	0.342	0.059

*ED,* emergency department.

**Table 5:** Association of intervention with patient satisfaction outcomes.

	No intervention (n=231)	Intervention (n=243)	P value	Mixed effects P-value*
% Acknowledge by patient name (n)	93.1 (215)	92.6 (225)	0.839	0.858
% Introduce (n)	96.1 (223)	97.9 (237)	0.244	0.318
% Explain role (n)	85.3 (198)	92.2 (224)	0.018	0.304
% Explain steps (n)	88.4 (205)	83.2 (198)	0.109	0.453
% Additional providers (n)	88.4 (205)	84.8 (206)	0.252	0.537
% Estimate duration (n)	11.3 (26)	13.1 (31)	0.558	0.647
% Return to ED (n)	94.4 (219)	95.5 (232)	0.592	0.595
% Refer friend to ED (n)	94.8 (220)	96.7 (235)	0.308	0.315
% Overall skill excellent (n)	82.3 (191)	87.7 (213)	0.104	0.110
Mean # CAT items excellent (SD)	12.3 (3.3)	12.7 (2.8)	0.184	0.238

*ED,* emergency department; *SD,* standard deviation.

* Mixed effect model only contained a fixed effect for intervention group and a random effect for student.
