# Supplementary material for: Effect of an Educational Intervention on Medical Student Scripting and Patient Satisfaction: A Randomized Trial
Source: West J Emerg Med. 2018 Mar 8;19(3):585–92. doi: 10.5811/westjem.2018.1.35992 (PMC5942029; doi:10.5811/westjem.2018.1.35992)
Supplement: Supplementary file 1 [file wjem-19-585-s001.docx]

Appendix A - Survey For Patients Who Opt In

1. Does your interaction with the medical student make you more likely to choose this emergency department in the future?

Y N

1. Does your interaction with the medical student make you more likely to refer a friend or loved one to this emergency department?

Y N

1. How would you rate the student’s skills at taking a history?

Poor 1 2 3 4 5 Excellent

1. How would you rate the student’s skills at doing a physical exam?

Poor 1 2 3 4 5 Excellent

1. How would you rate the student’s overall communication skills?

Poor 1 2 3 4 5 Excellent

How well did the medical student do in the following areas:

1. Greeting you in a way that made you feel comfortable?

Poor 1 2 3 4 5 Excellent

1. Treating you with respect?

Poor 1 2 3 4 5 Excellent

1. Showing interest in your ideas about your health?

Poor 1 2 3 4 5 Excellent

1. Understanding your main health concerns?

Poor 1 2 3 4 5 Excellent

1. Paying attention to you?

Poor 1 2 3 4 5 Excellent

1. Letting you talk without interruption?

Poor 1 2 3 4 5 Excellent

1. Giving you as much information as you wanted?

Poor 1 2 3 4 5 Excellent

1. Talking in terms you could understand?

Poor 1 2 3 4 5 Excellent

1. Checking to be sure you understood everything?

Poor 1 2 3 4 5 Excellent

1. Encouraging you to ask question?

Poor 1 2 3 4 5 Excellent

1. Involving you in the discussion as much as you wanted?

Poor 1 2 3 4 5 Excellent

1. Discussing the next steps?

Poor 1 2 3 4 5 Excellent

1. Showing care and concern?

Poor 1 2 3 4 5 Excellent

1. Spending the right amount of time with you?

Poor 1 2 3 4 5 Excellent

1. Performing any procedures (record only if a procedure was done)?

Poor 1 2 3 4 5 Excellent
