# Supplementary material for: Effect of an Educational Intervention on Medical Student Scripting and Patient Satisfaction: A Randomized Trial
Source: West J Emerg Med. 2018 Mar 8;19(3):585–92. doi: 10.5811/westjem.2018.1.35992 (PMC5942029; doi:10.5811/westjem.2018.1.35992)
Supplement: Supplementary file 2 [file wjem-19-585-s002.docx]

Appendix B – Data Collection Sheet

1. Student Name ____________
2. Student ID# ____________
3. ED Site where student is rotating _____________
4. Time Initial Observation Begins____________
5. Time Initial Observation Ends_____________
6. Time of Survey____________

Observation Data Points

1. Did the student wash their hands before entering the room?

Y N

1. Did the student acknowledge the patient using the patient’s name?

Y N

1. Did the student introduce himself/herself by name?

Y N

1. Did the student explain his/her role as a medical student?

Y N

1. Did the student ask any specific details about the patient’s complaint?

Y N

1. Did the student ask about the patient’s medications?

Y N

1. Did the student ask about the patient’s allergies?

Y N

1. Did the student ask about past surgeries?

Y N

1. Did the student ask if the patient uses tobacco?

Y N

1. Did the student ask how much alcohol the patient drinks?

Y N

1. Did the student listen to the patient’s lungs?

Y N

1. Did the student palpate the patient’s abdomen?

Y N

1. Did the student look in the patient’s throat?

Y N

1. Did the student look in the patient’s ears?

Y N

1. Did the student explain some of the steps (including diagnostic testing, medication administration, or observation) that would be used to address the patient’s complaint?

Y N

1. Did the student explain that additional providers (such as a resident or attending physician) would also be evaluating the patient?

Y N

1. Did the student offer an estimated duration of time that the patient would spend in the ED?

Y N

1. Did the student thank the patient for coming in?

Y N

1. Did the student order any tests prior to staffing with the attending physician?

Y N

1. Did the student order any medications prior to staffing with the attending physician?

Y N

1. Did the student perform any procedures on the patient?

Y N

1. Did the student call any consults on the patient?

Y N

1. Did the student go back and talk to the patient prior to discharge or admittance?

Y N
