# Supplementary material for: Effect of an Educational Intervention on Medical Student Scripting and Patient Satisfaction: A Randomized Trial
Source: West J Emerg Med. 2018 Mar 8;19(3):585–92. doi: 10.5811/westjem.2018.1.35992 (PMC5942029; doi:10.5811/westjem.2018.1.35992)
Supplement: Supplementary file 3 [file wjem-19-585-s003.docx]

**Appendix C – Interrater agreement between observers 1-4 and observer 5 (criterion standard)**

|  | **Observer 1** | | **Observer 2** | | **Observer 3** | | **Observer 4** | |
| --- | --- | --- | --- | --- | --- | --- | --- | --- |
|  | **Kappa** | **% Agree** | **Kappa** | **% Agree** | **Kappa** | **% Agree** | **Kappa** | **% Agree** |
| Acknowledge | 1.000 | 100.0 | 1.000 | 100.0 | 1.000 | 100.0 | 0.903 | 96.8 |
| Duration | 0.931 | 96.8 | 0.793 | 90.3 | 0.860 | 93.5 | 0.786 | 90.3 |
| Explain | 0.844 | 93.5 | 0.919 | 96.8 | 0.775 | 90.3 | 0.688 | 87.1 |
| Introduce | 0.912 | 96.8 | 0.912 | 96.8 | 0.912 | 96.8 | 0.712 | 90.3 |
| Other Providers | 0.832 | 93.5 | 0.843 | 93.5 | 0.919 | 96.8 | 0.919 | 96.8 |
| Role | 1.000 | 100.0 | 1.000 | 100.0 | 1.000 | 100.0 | 0.844 | 93.5 |
| Overall | 0.928 | 96.4 | 0.925 | 96.2 | 0.943 | 97.1 | 0.882 | 94.1 |
